# Student hostages in culture war

**DOI:** 10.1038/s44319-025-00527-0

**Published:** 2025-07-31

**Authors:** Bernd Pulverer

**Affiliations:** European Molecular Biology Organization, Meyerhofstrasse, Heidelberg, 69117 Germany

**Keywords:** Careers, Economics, Law & Politics

## Abstract

The US administration has extended its line of attack on academia from US federal research to private universities. The resulting abuse of international students is both ethically wrong and ultimately self-defeating.

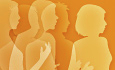

## Teaser

The US administration has extended its line of attack on academia from US federal research to private universities. The abuse of international students is both ethically wrong and ultimately self-defeating. The impact on scientific cooperation will be dire and threatens the standing of the USA as a nodal point for academic research.

The economic, cultural, and academic prowess of the USA has relied on its ability to attract international talent to create a uniquely fertile ground for creativity and innovation. The underpinning of this attraction has been a free and meritocratic environment that values dynamism, risk-taking, and low hierarchies. The US success story has until now been the envy of many; it seemed inimitable, buttressed by the country’s long-standing reputation as a beacon of academic freedom and stability, along with unparalleled public, private, and philanthropic funding opportunities. The global leadership in academia drives a formidable innovation economy, which in return supports academia, a positive feedback loop that seemed highly resilient to political change.

The pre-eminence of the USA in biomedical research could not, however, exist in isolation; it has been inexorably linked to the global academic enterprise. With the exception of sensitive data such as dual-use research, any policy that impedes the international exchange of data, reagents, infrastructure and, crucially, human capital will undermine this leadership position. Visa policies for longer-term positions have always been difficult to navigate for “alien” scientists (Elias et al, 2024), but the prospect of a world-class research environment with unparalleled funding still provided an irresistible lure. As a result, a disproportionate number of leadership positions in US academia and industry are currently populated by first-generation immigrants. China, a scientific superpower which already has overtaken the USA in a number of performance measures (Editorial, 2025), has realized this and engages proactively in international exchange—possibly encouraged by the prospect of stepping into a void left by a US administration apparently determined to thwart academic institutions in the USA.

During the past 6 months, the US administration and its supporters have made considerable efforts to undermine academic research and education with devastating effect. While the actions were foreshadowed by campaign promises and manifestos, even die-hard cynics would not have predicted the speed and breadth of the changes. Surprisingly, these actions appear to go far beyond an “America first” narrative to directly attack the country’s own leading seats of learning, which also happen to be some of the most globally recognizable and influential US brands.

The leveraging of strong-arm tactics such as attempts to block federal funding and licenses to award degrees to several public and private universities underscores how reckless this attack has become. The indiscriminate application of “efficiency measures”, arbitrary caps to overheads (Kamerlin and Elias, 2025), and erosion of academic independence already compromise the work of the NIH, the world’s biggest biomedical funder, its institutions and its fundees, as well as the NSF, which is on the verge of being expelled from its headquarters. Moreover, current budget proposals before the US Congress call for cuts of nearly 40% to the NIH and 55% to the NSF budget. The massive cuts that are already in effect compromise not only scientists, their host institutions, and international collaborative research, but also key infrastructures that global research relies on, including NCBI databases, necessitating the urgent discussion on better resilience of key infrastructures (Valencia, 2025).

Beyond the challenge to academic institutions, the most troubling turn of events from an international perspective is the undermining of scientific advice mechanisms on medical regulation alongside the partial defunding of infectious-disease research and public heath implementation via USAID and NIH international “subaward” grants (although the latter may now be averted) and, most importantly, by calling into question long established vaccinations (Caplan, 2025). If enacted, this will inevitably increase global mortality from infectious diseases in the US and elsewhere as controlled diseases re-emerge; it is not clear yet who is prepared to step in to continue public-health efforts in the global south, but it is laudable that some US institutions are rising to the challenge by continuing independent vaccine advice.

The tactics used by the government have proven effective: while many individual cases are subject to challenge in court, the multimodal attack on academia has saturated the legal system, risking to knock the much-touted “balance of power” off kilter. But it is also far from clear how far the courts will go, especially after the US Supreme Court overturned lower courts in allowing the deportation of migrants to third countries, and in light of personal attacks on lawyers and judges who have not fallen in line with government policy. The perceived threats to personal careers and the risk of direct attacks, such as the attempts to defund and de-license Harvard and Columbia University, have led to an eerie silence or compliance characterized by Philip Ball as ‘anticipatory obedience’ with telling historical antecedents (Ball, 2025).

The detention of several academics during regular travel suggests that international scientists are now likely to run a risk-benefit calculation before attending conferences in the USA, or elsewhere, if they are based in the USA. Conferences, the institutions they support, and scientific cooperation are likely collateral damage. In the face of compromized funding and rapidly increasing red tape that threatens to undermine academic freedom (Pulverer, 2025), the balance will be ever more likely to tip towards looking elsewhere for positions, which will exacerbate already falling US postdoc numbers. The academic powerhouses of Asia and Europe are only too happy to step in to benefit. But the next generation of international talent will also be considering their options as the magic pull of the USA as a career catalyst diminishes. The best have choices—why would they risk restricted travel options, harassment by government agents in public spaces, the premature termination of their studies or their research projects on account of rescinded permits or insecure funding? The economic cost to the USA will be severe - the government appears to accept the hit as it furthers its goal to weaken universities.

The question is by now academic for many, as after a pause to all new visa interviews, students from 25 countries are subject to outright visa bans, while systematic social-media screening to uncover content deemed “threatening to national security” -a term that can be interpreted in ambiguous ways - has been extended to all applicants. For many students, in particular those on the verge of graduation, the American dream will be turning into a nightmare. Even if they are not directly affected, there is the latent stress that they might be at any time and in any number of ways, be it through loss of visa, funding, or personal freedom. None of this is permissive for a creative research environment. Taking students hostage is unacceptable, and the global community should rise to their defense by providing alternatives for study, for degrees, and for planned or ongoing research to those who have been unfairly denied the education they paid dearly for. It is crucial to ensure ongoing projects can be continued with the same first authors elsewhere and that careers are thus unblocked—this is the only ethically defensible way out. We hope PIs can leverage their networks to find solutions.

Only three years ago, a commentary in this journal lamented the state of academic freedom in countries like Iran and Turkey, concluding: “as a global community, we scientists must not be silent in the face of this injustice. None of us assumes that our professional choice is a dangerous one, but far too many are a target of arrest, persecution, and in some cases death simply for being scientists.” (Kamerlin, 2022). It is tragic that the same statement can now be applied with some justification to the USA. Other countries must resist any populist tendencies to follow suit (Kamerlin 2025). 

## References

[CR6] Ball P (2025) Scientific institutions have a long history of anticipatory obedience. https://www.chemistryworld.com/opinion/scientific-institutions-have-a-long-history-of-anticipatory-obedience/4020931.article

[CR2] Caplan A (2025) Fool’s-gold science: The ethical and scientific perils of testing most vaccines using placebo-controlled randomized trials. EMBO Rep. 10.1038/s44319-025-00530-5 10.1038/s44319-025-00530-5 PMC12373718 40745394

[CR3] Elias MH, Fernández FM, Kamerlin SCL (2024) The ineligibility barrier for international researchers in US academia. EMBO Rep 25:457–458 38263328 10.1038/s44319-023-00053-x PMC10897176

[CR4] Kamerlin L, Elias MH (2025) NIH’s 15% cap: a cost comparison and research outlook. EMBO Rep 26:1676–1678 40102587 10.1038/s44319-025-00418-4 PMC11977179

[CR5] Kamerlin SCL (2022) Scholars in peril: when being a scientist can land you in jail (or worse). EMBO Rep 23:e56419 36373797 10.15252/embr.202256419 PMC9724652

[CR9] Kamerlin SCL (2025) Students must not be collateral damage in immigration clampdowns. 10.1038/s44319-025-00531-4 10.1038/s44319-025-00531-4 PMC12373955 40745393

[CR1] Editorial (2025) In science’s new era, open and transparent cooperation remains key. Nature 642:541–542 10.1038/d41586-025-01881-8 40527999

[CR7] Pulverer B (2025) Under pressure. EMBO Rep 26:1673–1675 40102588 10.1038/s44319-025-00419-3 PMC11977263

[CR8] Valencia A (2025) Decentralized databases in biomedical research: lessons from recent events. EMBO Rep 26:1679–1681 40102589 10.1038/s44319-025-00417-5 PMC11977236

